# Prediction of placebo responses: a systematic review of the literature

**DOI:** 10.3389/fpsyg.2014.01079

**Published:** 2014-10-01

**Authors:** Bjoern Horing, Katja Weimer, Eric R. Muth, Paul Enck

**Affiliations:** ^1^Department of Internal Medicine – Psychosomatic Medicine and Psychotherapy, University Hospital TübingenTübingen, Germany; ^2^Department of Psychology, Clemson UniversityClemson, SC, USA

**Keywords:** placebo, placebo response prediction, pain, optimism, self-efficacy, personality

## Abstract

**Objective:** Predicting who responds to placebo treatment—and under which circumstances—has been a question of interest and investigation for generations. However, the literature is disparate and inconclusive. This review aims to identify publications that provide high quality data on the topic of placebo response (PR) prediction.

**Methods:** To identify studies concerned with PR prediction, independent searches were performed in an expert database (for all symptom modalities) and in PubMed (for pain only). Articles were selected when (a) they assessed putative predictors prior to placebo treatment and (b) an adequate control group was included when the associations of predictors and PRs were analyzed.

**Results:** Twenty studies were identified, most with pain as dependent variable. Most predictors of PRs were psychological constructs related to actions, expected outcomes and the emotional valence attached to these events (goal-seeking, self-efficacy/-esteem, locus of control, optimism). Other predictors involved behavioral control (desire for control, eating restraint), personality variables (fun seeking, sensation seeking, neuroticism), or biological markers (sex, a single nucleotide polymorphism related to dopamine metabolism). Finally, suggestibility and beliefs in expectation biases, body consciousness, and baseline symptom severity were found to be predictive.

**Conclusions:** While results are heterogeneous, some congruence of predictors can be identified. PRs mainly appear to be moderated by expectations of how the symptom might change after treatment, or expectations of how symptom repetition can be coped with. It is suggested to include the listed constructs in future research. Furthermore, a closer look at variables moderating symptom change in control groups seems warranted.

## Background

Differences in placebo responsiveness have long been acknowledged and the topic of much empirical scrutiny. Since the onset of dedicated placebo research, the question of who does or does not respond to sugar pills has been discussed (for reviews, see Bootzin and Caspi, 2002; Kaptchuk et al., 2008). For some time, responding to placebos was considered a stigma for various reasons (Parkhouse, 1963; Brody and Brody, 2000). However, since the grounding of placebo responses (PRs) in a less mentalistic and more physiological framework—e.g., with the seminal work of Levine et al. (1978), involving the endogenous opioid system in placebo analgesia –, this stigmatization has partially subsided, at least in academia (Thompson et al., 2009). In recent years, especially with the widespread use of technology enabling the monitoring of brain activity, the responder question has been reconciled with mechanistic approaches. Consequently, interest in prediction of PRs was revived (Geers et al., 2005a) and since sparked a number of dedicated studies. Until recently however, the evidence supporting PR prediction was still considered disparate (Kaptchuk et al., 2008; Benedetti, 2009). A better understanding of placebo mechanisms, and therefore predictability of PRs, will allow for both the maximization and minimization of PRs. In established treatments, maximizing the additional PR is desirable. However, in early phases of pharmacological research, minimization and control of PRs is an important issue, in order to isolate and prove that a new treatment is effective beyond placebo (Enck et al., 2013).

This review is concerned with collating studies of sufficient methodological quality that address prediction of the PR. Terminological uncertainties notwithstanding (Hróbjartsson, 2002), we regard the PR as a symptom improvement caused by a placebo treatment and the context in which it is delivered. This causal relation is considered to be mediated mostly by expectancy and related mechanisms (Finniss et al., 2010; Enck et al., 2013). The core question to be addressed is: “Are the determinants of placebo responsiveness located within or outside of the individual?”

Placebo responsiveness could constitute a *stable* trait (uni- or multidimensional, e.g., a personality variable or genetic polymorphism). It would affect PRs independently of situational variables. People could therefore be classified on a dimension ranging from “responder” to “non-responder.” This point of view has been especially prevalent during early investigations into placebo responsiveness, but results have been inconclusive (Gryll and Katahn, 1978; Bootzin and Caspi, 2002).

To the contrary, the working model of most clinical trials—but also of many dedicated placebo studies—pragmatically emphasizes the fluctuating, *situational* determination of PRs. In fact, this is the implicit default assumption when determinants of PRs are not further considered in a study. Situational determinants can be generated outside (e.g., experimental manipulation) or inside the individual (e.g., mood). In this model, everybody is a potential responder, placebo responsiveness is ubiquitous and activated by situational influences (Bootzin and Caspi, 2002).

These simple explanations have merit to a certain degree: On the one hand, temperament, personality traits etc. by definition produce consistent behavioral output even in changing environments (Rothbart, 2007). On the other hand, sufficiently strong situations do have the ability to normalize a range of behaviors (Snyder and Ickes, 1985). However, most psychological characteristics conform to a more interactional view (David et al., 1997). This is reflected in the third basic explanation, which emphasizes how different trait variables *interact* with different situational variables to explain the extent of placebo responsiveness (Geers et al., 2007). Frequently, the focus of these approaches in placebo research lies on the therapeutic relationship and the quality of the interaction (Shapiro, 1971; Frank and Frank, 1973; Gryll and Katahn, 1978).

The latter concept can be elaborated by pointing out how PRs feed back to influence their determinants in future instances. This emphasizes possible changes in responsiveness over time and thereby the relevance of medical experience and socialization (the long-term dispositional and situational requirements to be met for placebo responsiveness to develop) (Moerman, 2000).

### Deduction of inclusion criteria

In the following, the rationale for inclusion of studies in the review is described. We use the term “predictor” to describe moderating variables measured prior to the placebo instruction, which are able to predict the response to placebo at a later time. In other words, we are solely looking at determinants of placebo responsiveness. Variables influenced by the placebo instruction (mediators or “proximal factors” such as expectancy, Vase et al., 2003) are not considered predictors along these lines.

#### Methodological inclusion criteria for valid statements concerning prediction of placebo effects

Not all study designs or data analyses permit internally valid statements about predictors of PRs. For example, the widely used cross-over design has been criticized on various grounds. In a cross-over design, all subjects are sequentially exposed to multiple or all experimental conditions; often, the design is counterbalanced, meaning that all permutations of condition orders are included (Grizzle, 1965). While generally advantageous in terms of statistical power, cross-over designs are prone to carry-over effects—even in counterbalanced designs, where asymmetric skill transfer can occur (Poulton and Freeman, 1966). Furthermore, large within-subject variability (as has been reported in placebo effects) and possible behavioral conditioning jeopardize internal validity (Enck et al., 2013).

Several design aspects enhance internal validity by abolishing many of the PRs' confounders; the guiding principle being how to assess the symptom change that would have happened in an untreated group anyway, simply by virtue of time, study participation, etc. (Hróbjartsson et al., 2011). Only studies including one or more of the following aspects will be considered sufficient to contribute to the responder discussion:

- *No-treatment control group*, a group receiving no treatment at all, but an otherwise identical protocol. Despite frequently being the best option, the certainty of not receiving anything leads to a mindset quite different from the placebo group, reducing actual comparability (Hróbjartsson, 2002)—the no-treatment group cannot be blinded. Within the scope of clinical trials, ethical and pragmatic reasons apply why a no-treatment control (“natural history”) is rarely possible (Benedetti, 2009).
- *“Open label” control group*, where participants are being applied the placebo, but are correctly informed that it is only an inert treatment. This approach has the shortcoming that an instruction of inertness does not necessarily induce the expectation of inertness—a participant might question whether the instruction is true and suspect the possibility that he or she is actually being deceived. Furthermore, conditioning mechanisms could be triggered regardless of information.
- While not strictly a design aspect, a *known natural course of symptoms* amounts to an effective control condition (Benedetti, 2009). For example, knowing that symptoms in a specific medical condition either remain constant or worsen, any significant improvement in the placebo group would at least preclude spontaneous remission. However, to “know” a course requires having measured it at some time, therefore constituting a historical control group likewise exposed to all biases inherent in study participation. Caution has to be taken not to underestimate the singular conditions of each study in terms of sample, setting etc. (Hróbjartsson, 2002).
- *Different levels of placebo manipulations*. While not permitting conclusions regarding the absolute size of PRs, different levels of independent placebo variables arguably permit relative statements, e.g., “two pills are more effective than one.” Therefore, the interaction between predictors of interest and the placebo manipulation can be examined.

#### Statistical inclusion criteria for valid statements concerning prediction of placebo effects

**Figure 1:**
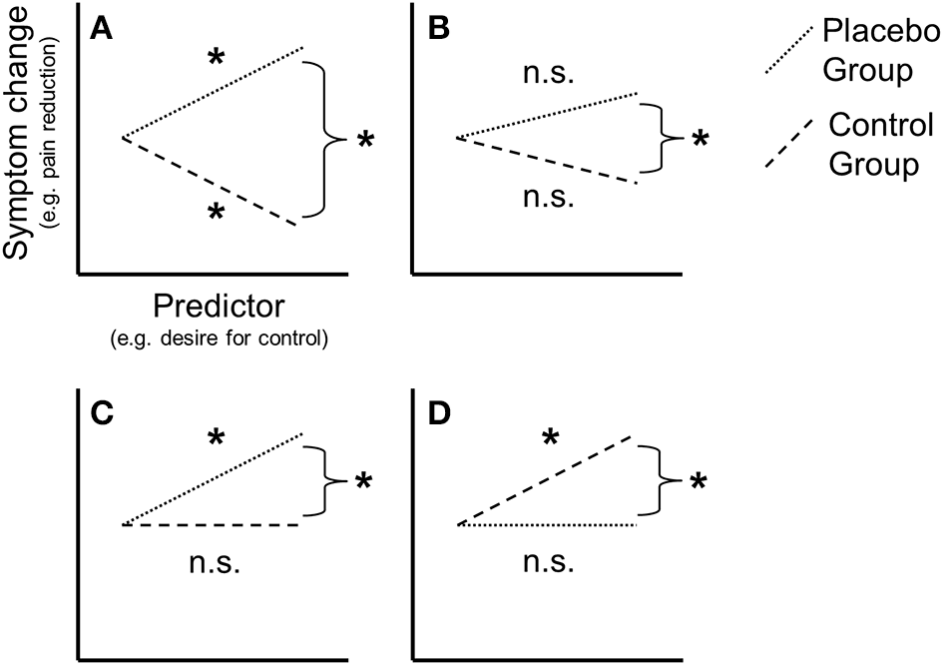
**Conceptual illustration of some possible interactions between predictor(s) and symptom change in placebo group (PG) and control group (CG)**. Each regression slope represents a correlation between a predictor (x-axis) and symptom change (y-axis) in either PG or CG. The single regression slopes can be either significant (as denoted by the asterisk) or not significant (n.s.). For valid placebo response prediction, the two slopes must be significantly *different*, as denoted by the asterisk next to the curly brackets. **(A)** *Differential association* of predictors and symptom change in PG and CG (both correlations significant but opposite, e.g., positive in PG, negative in CG). **(B)** *Subthreshold association* (no correlation significant, only slope difference). **(C)** *Placebo-association* (correlation significant in PG but not CG). **(D)** *Placebo-disassociation* (correlation significant in CG but not PG).

If an adequate control condition has been established, it has to be duly considered in statistical analysis. Not all studies fulfill this requirement, e.g., when predicting PRs only in the placebo group, without considering the no-treatment control group in the same regression (Lyby et al., 2010). In analogy to the determination of the extent of PRs (which can only be achieved by comparison to an adequate control), this tells us little: After all, the correlation of predictors and symptom change (pre- to post-placebo) could be entirely due to natural history and various other effects of measurement repetition. Even if the correlation is significant in one group but not the other (e.g., Fillmore et al., 1998), it is not guaranteed that there is a significant difference between the two correlations. This would have to be established by methods such as Fisher's *Z*-test or regression analysis. Therefore, analyses specifically aimed at PRs have to show *differential* prediction of symptom change in placebo vs. control. Figure 1 shows the relevance of examining this interaction. Once differential prediction is established, the assessment allows for determination of whether the correlation is divergent in both groups (A, B) or actually gets associated (C), or dissociated (D) in the placebo group (for an applied example, see Figure 1 of Handley et al., 2011). In particular, establishing differential prediction allows identification of otherwise “silent” effects, when no significant correlations exist in either placebo or control group (B).

## Materials and methods

### Search strategy and categorization of studies

Two independent search strategies were used to identify studies investigating determinants of placebo responsiveness. The first search was performed in a database established in 2004 by our group. At the inception of this database, about 100.000 citations from PubMed were retrospectively examined for dedicated treatment of the PR by using the search string “placebo.” Excluded were “randomized placebo-controlled trials…, editorials, letters to the editor and review papers and meta-analyses not related to the PR *per se*” (Enck et al., 2011). From 2004 on, new articles were screened on a weekly basis. In January 2014, the database contained 2540 dedicated placebo publications. The database mainly contains publications researched via PubMed, published in English, between 1953 and January 2014; however, papers from numerous other sources are included.

**Figure 2:**
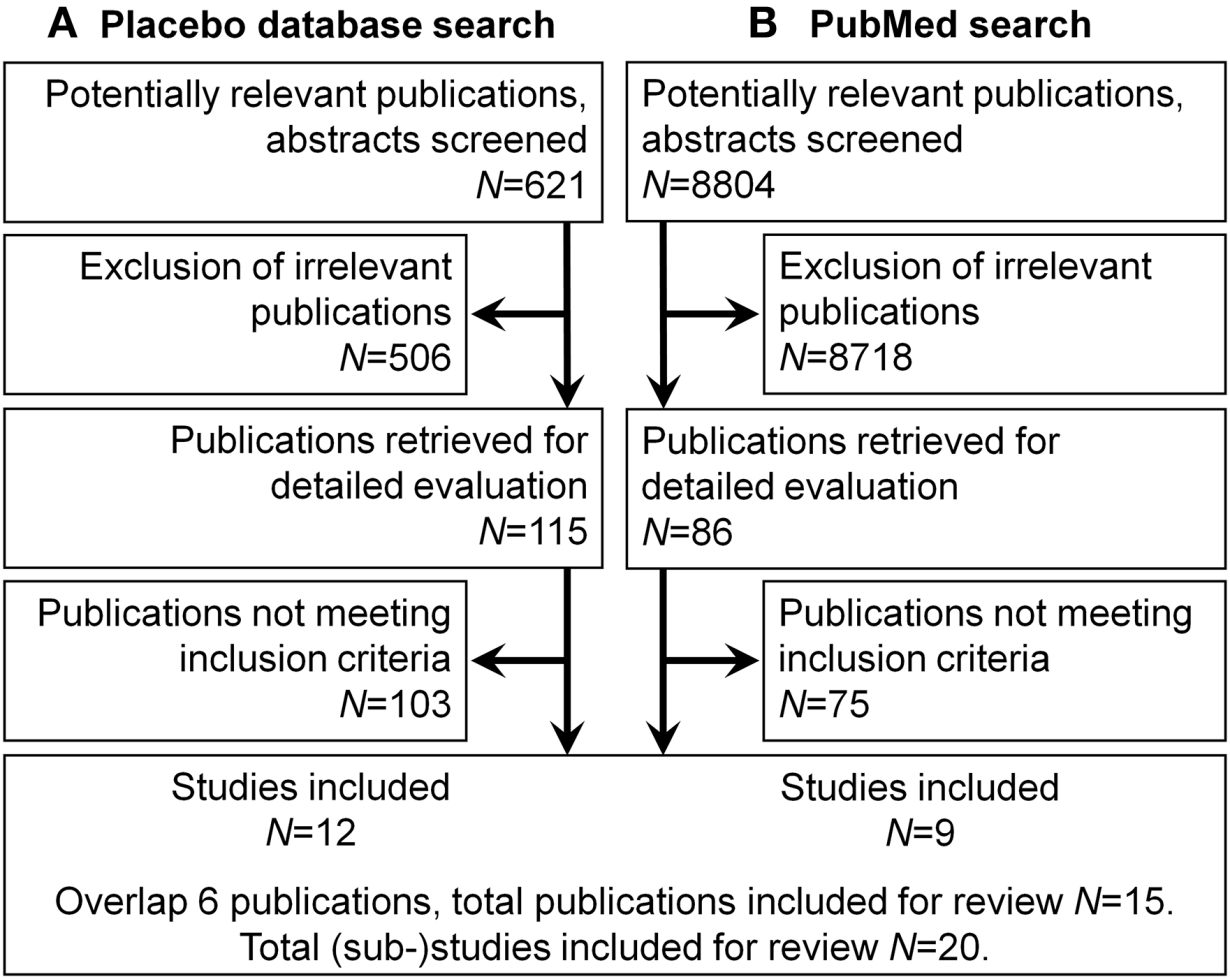
**Flow diagram of study selection**. A total of 15 publications were included containing 20 (sub-)studies. **(A)** Placebo database search. **(B)** Pubmed search.

Relevant publications were identified using methodological descriptors (in title, abstract, or MeSH terms) indicative of responder analyses such as “correl^*^”, “predict^*^”, “reactor”, “regress^*^”, or “respond^*^”. After accounting for duplicates, the database search yielded *N* = 605 publications. Via unsystematic search of bibliographies and from various other sources, an additional 16 publications were identified for screening, totaling *N* = 621 (Figure 2A).

Of these publications, abstracts were examined and all but original data papers were excluded. In detail, publications obviously not involving responder analyses were not considered, nor were reviews, meta-analyses, or concept papers. For conceptual clarity, nocebo studies were excluded as well, notwithstanding the possible allocation of both placebo and nocebo on a single dimension (Petrovic, 2008; Scott et al., 2008). Furthermore, correlates of PRs were excluded if no temporal sequence of predictor and criterion was established by study protocol, i.e., if the putative predictor was assessed after the placebo instruction. For example, this concerns expectancy, or differences in endocrinological or neuronal activation concurrent with the PR (Whalley et al., 2008; Ober et al., 2011).

Of the subsequently identified 115 publications, Methods and Results Sections were screened for adequacy in regard to the methodological inclusion criteria outlined above, resulting in further reduction to 32 articles. In the final step, statistical inclusion criteria were applied and *n* = 12 publications remained (see Figure 2A).

The second search strategy was performed in PubMed. It used an exhaustive search string modified from Hróbjartsson and Gotzsche (2010) on English or German entries published before February 2014. This string included general search terms as well as MeSH terms, and was structured in three major segments:

- Segment one referred to “placebo” and variations like “sham”
- Segment two referred to data structure, statistical and methodological aspects like “association,” “correlation,” or “no-treatment”
- Segment three referred to various exclusion criteria such as non-original articles and animal experiments.

Furthermore, due to the large number of hits (>100.000), only publications including “pain” or related terms like “analgesia” were considered. The complete search strategy can be obtained from the authors. Pain was chosen specifically because of the central role it has for placebo research (Hoffman et al., 2005): Not only does it have the largest empirical basis, but the methodologically soundest research has been considered to come from this area.

The search yielded *N* = 8804 publications (Figure 2B). Abstracts were examined one by one and a checklist for inclusion was applied. Non-inclusions were tagged with justifications for exclusion; since multiple tags could be assigned, there is a minor overlap in exclusion categories. All dedicated placebo research papers were included by default for the possibility of secondary predictor analyses not mentioned in the abstract, except for 154 articles where it was clear from the abstract that a cross-over design or no adequate control was used. Most excluded studies (*n* = 7717) were tagged as clinical studies which mentioned placebo only as a control condition. 410 articles were excluded for publication type (e.g., reviews, editorials). 459 studies were excluded for study sample (e.g., animal research).

Selection resulted in *n* = 86 publications overall. Of these, Methods and Results Sections were screened according to the above inclusion criteria. 42 were excluded by virtue of not analyzing potential predictors mentioned in the abstract; 12 were excluded due to not meeting methodological inclusion criteria; 22 for not meeting statistical inclusion criteria; 1 article could not be obtained. After experimental design and statistical analysis were taken into account, 9 publications remained.

With an overlap of 6 publications, the second search contributed 3 publications for a total of *n* = 15 considered in the review. In these publications, a total of 20 (sub-)studies were described and suited for predictor analysis (Tables 1, 2).

**Table 1:** **Studies investigating placebo response prediction in pain**.

**References (sub-study)**	***N* (f:m) population**	**Symptom or variable**	**Placebo response predictor(s)**	**Category**
Bjorkedal and Flaten, 2011	23 (7:16)	Pain^a^ intensity	[No significant predictors]	
Bjorkedal and Flaten, 2012	72 (36:36)	Pain^b^ intensity	Female sex	Trait/stable
De Pascalis et al., 2002	72 (47:25)	Pain^c^ intensity	Match of suggestion and suggestibility (SSS)	Interactional
Geers et al., 2010	116 (60:56)	Overall pain^d^	Higher trait optimism (LOT-R)	Trait/stable
		Pain^d^ severity	Higher trait optimism (LOT-R)	Trait/stable
		Pain^d^ intensity	Higher trait optimism (LOT-R)	Trait/stable
Geers et al., 2013-1	94 (62:32)^*^	Pain^d^ intensity	Higher habitual desire for control (DCS)	Trait/stable
Hall et al., 2012	112 (83:29)	Pain^e^ and other symptoms	Increased dopamine availability due to methionine alleles	Trait/stable
	IBS patients			
Handley et al., 2011	49 (23:26)	Pain^d^ intensity	Lower belief in expectation biases (5-item scale)	Trait/stable
		Pain^d^ affect	Lower belief in expectation biases (5-item scale)	Trait/stable
		Systolic blood pressure	Higher belief in expectation biases (5-item scale) lead to decrease in systolic blood pressure	Trait/stable
Levine et al., 1979	107 (?:?)	Pain^f^ severity	Higher baseline symptom severity (VAS)	Situational
	OP patients			
Staats et al., 2001	48 (41:7)	Pain^d^ intensity	[No significant predictors]	

*Sub-studies are indicated by consecutive numbers as they appeared in the respective publications. Population consists of healthy students unless otherwise noted. Placebo response predictors have been tentatively categorized into trait/stable characteristic, situational characteristic or interactional characteristic. As interaction type according to Figure 1 could not be assessed from the majority of studies, no classification is performed. Example: Handley et al. (2011) investigated 49 healthy participants and found that higher PR in cold pressor pain intensity and pain affect was predicted by lower belief in expectation biases, and that decrease in systolic blood pressure was predicted by higher belief in expectation biases. Belief in expectation biases is considered a stable characteristic. Abbreviations: DCS, Desire for Control Scale; IBS, Irritable Bowel Syndrome; LOT-R, revised Life Orientation Test; SSS, Sensory Suggestibility Scale*.

* *Overlap between sub-study samples not clear*.

a-f *Pain protocols: ^a^Laser heat, ^b^Thermode heat, ^c^Electrical, ^d^Cold pressor, ^e^Clinical/symptomatic, ^f^Postoperative*.

**Table 2:** **Studies investigating placebo response prediction in symptoms other than pain**.

**References (sub-study)**	***N* (f:m) population**	**Symptom or variable**	**Placebo response predictor(s)**	**Category**
Brockner and Swap, 1983	30 (?:?)	Sleep onset (minutes; self-report)	Higher body consciousness	Trait/stable
	Insomniac students		Low self-esteem (revised Janis-Field Self-Esteem Scale)	Trait/stable
Darragh et al., 2014	60 (41:19)	Stress (heart rate change)	Higher fun seeking (BIS/BAS scale)	Trait/stable
			Higher sensation seeking (BIS/BAS scale)	Trait/stable
			Lower neuroticism (EPQ-R Short Version)	Trait/stable
Geers et al., 2005b-1	45 (21:24)^*^	Affect	Match of individual goals with possibility to achieve goal by confirming placebo expectation	Interactional
Geers et al., 2005b-4	57 (37:20)^*^	Caffeine symptoms	Match of individual goals with possibility to achieve goal by confirming placebo expectation	Interactional
		Systolic blood pressure	Match of individual goals with possibility to achieve goal by confirming placebo expectation	Interactional
Geers et al., 2005b-5	59 (35:24)^*^	Affect	Match of individual goals with possibility to achieve goal by confirming placebo expectation	Interactional
Geers et al., 2007	56 (38:18)	Sleep quality	Higher trait optimism (LOT-R)	Trait/stable
Geers et al., 2013-2	98 (67:31)^*^	Auditory discomfort	Higher habitual desire for control (DCS)	Trait/stable
Geers et al., 2013-3	121 (77:44)^*^	Auditory discomfort	Higher state desire for control (coding of written manipulation check score)	Situational
Heatherton et al., 1989-1	129 (129:0)^*^	Calorie intake	High restraint (Restraint Scale) in hungry-condition	Interactional
Heatherton et al., 1989-2	60 (60:0)^*^	Calorie intake	High restraint (Restraint Scale) in hungry-condition	Interactional
Horing, 2013	28 (15:13)	Motion sickness	Lower generalized self-efficacy (GSE)	Trait/stable
			Lower internal locus of control (FKK)	Trait/stable

*For table structure see caption of Table 1. Abbreviations: BIS/BAS, Behavioral inhibition system/behavioral activation system; CFC, Consideration-of-Future-Consequences scale; DCS, Desire for Control Scale; EPQ-R, revised Eysenck Personality Questionnaire; GSE, General Self-Efficacy scale; FKK, competence and control belief scales [Fragenbogen zu Kompetenz und Kontrollüberzeugung]; LOT-R, revised Life Orientation Test*.

* *Overlap between sub-study samples not clear*.

## Results

A general overview of the 15 articles is provided in Tables 1, 2. Sub-studies are indicated by consecutive numbers as they appeared in the respective publications, e.g., Heatherton et al. (1989-2) for the publication's second sub-study. Categorization into stable/situational/interactional predictors is tentative, since most psychological phenomena meet the criteria of more than one category. Nine studies concerned with the identification of PR predictors used experimental or clinical pain as a dependent variable (Table 1). Three studies used cardiac parameters; two studies each used affect, auditory discomfort, calorie intake and sleep parameters, while one study each used caffeine symptoms and motion sickness (Table 2). Overlap exists due to multiple outcome variables.

### Trait predictors of placebo responses

The statistical nature of the findings (being differentially predictive of PRs in the respective modality, as compared to an adequate control) is shortened as “predictive.” Eleven studies were identified which reported stable predictors.

Several psychological constructs predictive of PRs in various modalities were closely related. As proposed (Geers et al., 2005a), dispositional optimism significantly predicted placebo analgesia in Geers et al. (2010). Geers et al. (2013) reported that habitual desire for control predicted the PR on cold pain in sub-study 1, and the PR on auditory discomfort in sub-study 2. Furthermore, the investigators identified perceived stress as predictive for performance in a choice reaction time task. Darragh et al. (2014) recently reported a significant prediction of change in heart rate after placebo. Their regression model included positive interactions of group (placebo vs. control) with fun and sensation seeking, and negative interactions of group and neuroticism. In a *post-hoc* analysis of one of our own studies (Horing et al., 2013), we found an opposite effect of both self-efficacy and internal locus of control: Higher scores predicted symptom improvement in the control group but symptom worsening in the placebo group (Horing, 2013). Another experiment by Geers' group (Handley et al., 2011) found a lower belief in expectation biases to be predictive of PRs–in other words, participants who assumed expectation biases as less relevant concerning symptom reports were actually more influenced by placebo instructions. Lastly, Brockner and Swap (1983) found a higher body consciousness (a psychological construct containing the extent and precision of inward-directed attention), combined with low self-esteem, as predictive of a “reverse placebo effect” on insomnia. Study participants who were instructed that a pill had an arousing effect actually relaxed better and had shorter sleep-onset latency.

Putative biological markers identified for placebo responsiveness were participant sex and dopamine availability. In an unusual design where a placebo instruction was used to facilitate conditioned pain modulation, Bjorkedal and Flaten (2012) reported that only women responded with increased analgesia. In a study by Hall et al. (2012), increased PRs were associated with increased dopamine availability as indexed by methionine alleles of the val158met polymorphism.

### Situational and interactional predictors of placebo responses

In seven studies, five psychological predictors were identified with clear internal situational, or interactional characteristics.

Contingent on the experimental manipulation, trait optimism was found to significantly predict expectancy effects on sleep quality in Geers et al. (2007)—with a positive correlation following placebo instruction, and an inverse correlation following nocebo instruction.

Three of the five sub-studies reported by Geers et al. (2005b) could be included in the review, namely sub-studies 1, 4, and 5. They reported significantly increased PRs on various symptoms (affect, caffeine effects, blood pressure) when the participants were able to fulfill a goal (habitual or experimentally instilled) by the confirmation of the placebo expectation. For example, they used semantic priming to instill a motive for cooperation, which the compliance with the placebo instruction could fulfill. Geers et al. (2013-3) investigated PRs on auditory discomfort after desire for control was experimentally induced. The association between desire for control and the PR was only activated when there was a possibility to choose between the (factually equivalent and inert) treatments.

De Pascalis et al. (2002) found that suggestibility only exerted significant influence on the PR in the stronger of two expectation conditions; in the weaker condition, there was no difference between highly and lowly suggestible individuals. In a rather unusual design, Heatherton et al. (1989-1 and 2) examined whether calorie intake was differentially influenced by “placebo” instructions by dietary restraint (i.e., abnormally monitoring one's eating behavior or following certain dietary restriction). The instruction included the suggestion that participants either felt hungry or full. Indeed, placebo instructions only worked in the suggested direction in restrained eaters (eating more after the “hungry”-pill, and less after the “full”-pill).

Among symptom- and pathology-related predictors, Levine et al. (1979) reported a higher baseline pain severity as differentially associated with placebo analgesia; this is the only study which did not use a parametric outcome as dependent variable, but a dichotomous responder definition.

### Non-significant predictors of placebo responses

Negative results were more difficult to ascertain. Single parameters that were found not to be predictive of PRs in the respective studies were the Fear of Pain Questionnaire (Bjorkedal and Flaten, 2012), Pain Anxiety Symptom Scale (Staats et al., 2001), revised Life Orientation Test (Bjorkedal and Flaten, 2012; Horing, 2013) as well as Beck Depression Inventory, Proactive Coping Inventory, State-Trait Anxiety Inventory (all Horing, 2013), and participant sex (Bjorkedal and Flaten, 2011). When single parameters were mentioned, they were not or could not always be considered as predictors. Among those mentioned but not analyzed were the revised Self-Monitoring Scale (Handley et al., 2011), revised Life Orientation Test (Handley et al., 2011; Darragh et al., 2014), Consideration-of-Future-Consequences scale (Handley et al., 2011), and Tellegen Absorption Scale (Darragh et al., 2014). In addition to specific measures, three studies referred to “packets of prescreening questionnaires [completed] earlier in the semester” (Geers et al., 2010; Handley et al., 2011) or “a brief packet of questionnaires” (Geers et al., 2007).

## Discussion

The prediction of PRs has been an endeavor of investigators from the onset of dedicated research into the phenomenon. This review attempted to assess the state of knowledge by applying conservative methodological criteria for study inclusion. Surprisingly few original papers prevailed—all in all, only 20 (sub-)studies met the criteria. While it is possible that the search was overly conservative, the goal was to identify only those studies from which variables that differentially predicted symptom change in the placebo group vs. symptom change in the control group were clearly distinguishable.

Results are heterogeneous, but imply that predictors are rather not found among “classic” trait personality variables (such as extraversion or neuroticism) or emotional response dispositions (such as anxiety). Instead, they are more on the side of cognitive constructs such as self-efficacy, locus of control, and “emotionalized” contingency expectations. From a theoretical perspective, this is intuitive, considering how closely related these concepts are to expectancy (Bandura, 1977). Expectancy is considered a major PR mechanism (Finniss et al., 2010; Enck et al., 2013).

An important cluster seems to revolve around an internality-externality dimension, such that participants follow placebo instructions more readily when having an external locus of control. For example, results by Heatherton et al. (1989) have led the investigators to hypothesize that restrained eaters lack sensitivity to internal states and are therefore more reliant on external cues to regulate behavior. The “permission” implicit in the placebo instructions could provide such a cue. We propose that this readiness is actually tied to low self-esteem (Brockner, 1988), which would explain our own finding concerning predictors of PRs on motion sickness (Horing, 2013). This interpretation is supported by self-esteem moderation of PRs on sleep onset reported by Brockner and Swap (1983).

Many of the mentioned constructs also draw attention to potential sources of bias in repeated symptom measurements and emphasize the necessity of careful experimental design when investigating PRs. This notion is especially prevalent when considering results by Handley et al. (2011) involving the belief in expectation biases in consequent placebo analgesia.

Due to its broad psychological ramifications, sex seems a less tangible predictor of PRs. Debate is still open what relevance it has for placebo responsiveness (Aslaksen et al., 2011). Results presented here are mixed. Both psychological and biological explanations for the positive predictive value of sex are offered by Bjorkedal and Flaten (2012), albeit the paradigm used is rather unusual (if intriguing), limiting comparability. In general, it seems too convenient to restrict oneself to sex for psychologically codetermined response variables like pain. Instead, it seems worthwhile to include measures of (psychological) gender to explore sociocultural influences.

It has been noted that there is not just one PR affecting all symptom modalities, but many (Benedetti, 2009), potential overlap notwithstanding (Petrovic et al., 2005). This could explain some of the heterogeneity—for example, the involvement of optimism in pain and sleep quality (Geers et al., 2007, 2010), but not in stress or motion sickness (Horing, 2013; Darragh et al., 2014). This cautions one not to overgeneralize findings in single symptom modalities, but instead try to describe the qualitative characteristics of the investigated conditions. So far, the data base for a symptom-specific assessment is thin. Pain would be the most promising candidate for an isolated analysis.

As far as the characterization of placebo responsiveness as a trait or state variable is concerned, this review offers little to resolve the issue. We feel that a pattern is discernible which warrants further investigation of stable characteristics such as personality variables. This is in line with other studies in the field (Pecina et al., 2013) and does not contradict more interactional oriented approaches (Kong et al., 2013).

We are confident of the reliability of the selection method. Yet, this review has several limitations that need to be addressed. First, the sample of studies examined for inclusion can be criticized. Research has been mostly constrained to PubMed, resulting in a probable bias for medical literature; more psychologically oriented literature, as e.g., accessible via PsycInfo, was not included. This shortcoming has to be eliminated by a more thorough, dedicated research amounting to a systematic review or meta-analysis. Secondly, publication bias is an important issue in placebo research (Hróbjartsson et al., 2011) and certainly affects this review, as well. Presumably, negative results in particular will be underrepresented. Thirdly, inclusion criteria were rather conservative. Especially the exclusion of cross-over designs proved to be a severe restriction, prohibiting consideration of pertinent studies (e.g., Aslaksen et al., 2011; Pecina et al., 2013).

Yet, this concession is congruent with most authoritative sources conceding a scarcity of *reliable* data concerning predictors of placebo responsiveness (Kaptchuk et al., 2008; Benedetti, 2009). For example, Hall et al. (2012) implicated the reward system in placebo responsiveness. While they corroborated previous evidence (Leuchter et al., 2009), the latter publication could not be included due to lack of an adequate control. Another article carried similar implications by reporting an association of gray matter density in reward sensitivity related brain areas and PRs (Schweinhardt et al., 2009). Unfortunately, the study used a cross-over design which was not considered for inclusion in this review. Likewise, other studies did not have sufficient validity regarding reward sensitivity (de la Fuente-Fernandez et al., 2001; de la Fuente-Fernandez and Stoessl, 2002; Scott et al., 2007). Most relevantly, de la Fuente-Fernandez et al. (2001) and de la Fuente-Fernandez and Stoessl (2002) only assessed post-instruction associations of placebo responding with reward sensitivity. As to the first study (de la Fuente-Fernandez et al., 2001), sample size was very low (*N* = 6), thereby not granting sufficient protection against chance results; furthermore, carry-over effects seem likely, as far as the procedure could be ascertained. The second study did use a control group to assess novelty effects of first exposure to the stimulus material, but no control incorporating the effects of measurement repetition (de la Fuente-Fernandez and Stoessl, 2002). Scott et al. (2007) reported a correlation between Nucleus accumbens-activity in placebo analgesia, and after monetary reward. While the results are highly interesting and were well received, the investigators did not include an adequate control group. Therefore, it cannot be precluded that simple measurement repetition would have the same (or at least aligned) effects.

Pertaining to the explanatory value of optimism, some precautions apply to the generalizability of these findings. On the plus side, the effect has been analyzed with excellent methodology. However, results come only from a single research group (Geers and colleagues) and should be replicated elsewhere. Unfortunately, while Morton et al. (2009) investigated optimism as well, they only performed an isolated analysis of the placebo group. It should also be noted that other investigators found conflicting results compared to Geers' group. For example, one study did not replicate the interaction of optimism with a placebo instruction, but only a main effect of optimism (Hanssen et al., 2014). Relatedly, the induction of state optimism was found to decrease pain (Hanssen et al., 2013), even though no placebo was applied—effectively, this amounts to an optimism effect in the control group, which did not show in the aforementioned experiments. Darragh et al. (2014) did not find an association of optimism with the PR on physiological stress parameters, nor did we find an association with the PR on motion sickness (Horing, 2013).

A more basic criticism can be leveled at studies presented by Geers and colleagues. Apparently, the basic efficacy of their placebo paradigms has not been established—in three of five publications, main effects of condition (e.g., placebo vs. control) were not significant (Geers et al., 2005b, 2007, 2010). Considering that the efficacy of placebo has been established in many prior studies, it can be argued that a significant mean difference should constitute a benchmark below which the symptom change in the placebo group cannot be considered a PR.

On a more general note, the majority of studies were performed with healthy undergraduate students, limiting generalizability in terms of age, medical condition, and other parameters. Of the 20 studies reporting placebo predictors, nine were performed by the same research group. Lastly, it is not guaranteed that whatever predicts placebo responsiveness on one occasion will be predictive at another—this applies to questions of reliability, but also to the correspondence of psychological predictors to culturally engrained medical concepts (Walach and Jonas, 2004). Apart from methodological issues, this might be the reason why a number of variables (e.g., neuroticism) which have been found to be involved in placebo responsiveness in the 1950s and 60s have rarely been implicated since (but cf. Darragh et al., 2014).

One of the most intriguing notions of the presented research is the role of the control group in placebo experiments. A number of the constructs identified here warrant a deeper engagement with the nature of this experimental aspect, as has been discussed before, e.g., on the topic of bias correction (Handley et al., 2011). As another example, the induction of optimism can lead to significant analgesia in groups not receiving any treatment at all (Hanssen et al., 2013). The same applies for self-efficacy in motion sickness (Eden and Zuk, 1995; Horing, 2013). It may be that the question of placebo responsiveness is malformed: That it is not about identifying who responds best to placebo, but how to create a paradigm that best reinforces aspects like optimism and self-efficacy, treatment given or not. When members of the control group are being told “you will not get any treatment before the retest…,” it might as well imply, “… so however you deal with the symptoms at retest is entirely up to you.” It seems worthwhile to not only look at what happens in the group receiving the placebo treatment, but to take the time to examine why specific symptoms change in the control group, in order to figure out why placebos are effective at reducing those symptoms at all. Not the least due to ethical constraints, this genuinely psychosomatic question has only rarely been addressed.

To conclude, despite the repeated and widely received emphasis on the necessity of adequate controls (Hróbjartsson, 2002; Kaptchuk et al., 2008; Hróbjartsson et al., 2011), studies are often ill-suited to provide answers concerning predictors of the PR. Control groups are omitted either by design, or by insufficient consideration in statistical analysis. In the latter case, many studies could be revisited to perform *post-hoc* analyses on response prediction, particularly involving readily available parameters like sex. Keeping in mind the complex interactions of the concrete experimental designs with the respective predictors, a number of variables have been repeatedly involved. It seems worthwhile to include the identified variables in future studies—especially, independent replication of some of the more frequently mentioned parameters is needed. Specifically, promising variables are goal-seeking, self-efficacy, self-esteem, locus of control, and optimism; desire for control and restraint; fun and sensation seeking, and neuroticism; participants' sex (or possibly gender); the val158met-polymorphism; suggestibility, beliefs in expectation biases, body consciousness and baseline symptom severity. At this point, it seems feasible to employ confirmatory analyses involving a priori defined constructs and appropriate error correction.

### Conflict of interest statement

Work of the authors is supported by the Volkswagen Foundation Grant No. I/83 805 from Volkswagen Foundation (Hannover, Germany), a grant from the Deutsche Forschungsgemeinschaft (DFG En 50/30-1) within the DFG Centre Grant FOR 1328, and the Institutional Strategy of the University of Tübingen (DFG ZUK 63). The authors also acknowledge support by Deutsche Forschungsgemeinschaft and Open Access Publishing Fund of the University of Tübingen. The authors declare that the research was conducted in the absence of any commercial or financial relationships that could be construed as a potential conflict of interest.
